# Hermaphroditismus, Intersexualität, Zwitter, Varianten der Geschlechtsdifferenzierung – eine kurze Historie von Diskursen

**DOI:** 10.1007/s00120-021-01509-5

**Published:** 2021-04-12

**Authors:** Friedrich H. Moll

**Affiliations:** 1 Institut für Geschichte, Theorie und Ethik der Medizin, Heinrich-Heine-Universität, Düsseldorf, Deutschland; 2 Museum, Bibliothek und Archiv zur Geschichte der Urologie, Düsseldorf – Berlin, Deutschland; 3 Urologische Klinik, Kliniken der Stadt Köln gGmbH, Urologischer Arbeitsplatz, Krankenhaus Merheim, Neufelder Straße 32, 51067 Köln, Deutschland

## Re-Review zu

Margaux Becker V, Schneiders M, Hennecken M, Stolp S, Lenz J, Moll FH, Leißner J (2021) Junger Mann mit simultanem Unterbauch- und Hodentumor. 10.1007/s00120-021-01510-y

Der Begriff „Gender“ bzw. „soziales Geschlecht“ in Abgrenzung zum biologischen Geschlecht „sex“ als zentraler Begriff der Forschungen zu Geschlechtern wurde 1955 erstmals von dem Sexualwissenschaftler und klinischen Psychologen John Money (1921–2006) in einem Aufsatz über Hermaphroditismus verwendet [1]. Vorher waren die Bezeichnungen „sex role“ oder „sex identity“ gebräuchlich. Häufig werden auch heute noch wichtige Begriffe in Medizin, Psychologie, Gesellschaftswissenschaften, bei Betroffenen und Historie uneinheitlich verwendet, da „Intersexualität“ und der Umgang hiermit stark von den jeweiligen Normen einer Gesellschaft und den dazugehörigen Einstellungen zu Geschlecht und „geschlechtlichen“ Körpern abhängen [2].

Allein der Begriff „Intersex“ wird teilweise als „Identitätslabel“ oder als rein medizinische Zuschreibung im Rahmen eines euroamerikanischen Diskurses angesehen [3, 4].

Die Gestalt des „Hermaphroditos“ wie auch der Begriff „Hermaphrodit“ lassen sich bis zum 4. Jahrhundert vor Chr. zurückverfolgen, wenn auch schon vorher androgyne Gottheiten von östlichen Religionen in Griechenland wahrscheinlich bekannt gewesen waren [5].

Seit der frühen Neuzeit waren Hermaphroditen ein ideales Untersuchungsgebiet für Mediziner, um deren eigene Kompetenz und Zuständigkeit für geschlechtliche und sexuelle Fragen zu demonstrieren und Geschlechtsdefinitionen und Klassifikationen praktisch zu überprüfen und fortschreiben zu können [6]. Der geschlechtlich uneindeutige Körper wurde zum Problem. Zum Mythos des Hermaphroditen in den Bereichen Kunst und Literatur liegen vielfältige Studien vor [7–9].

Während bis zur Aufklärung die Beschreibung eher allgemein erfolgte, lassen sich mit dem Zeitalter der Aufklärung Abhandlungen finden, die dazu dienten, das wesensmäßige Geschlecht männlich oder weiblich herauszuarbeiten und das wahre Geschlecht zu finden [10]. Seit dem 19. Jahrhundert kennen wir systematische Fallberichte. Der Einzelfall sollte jetzt dazu dienen, das Wesentliche herauszuarbeiten [11]. Gleichzeitig setzte auch ein Problematisierungs- und auch Medikalisierungsprozess ein – das uneindeutige Geschlecht wurde zum Problem der Medizin [12].

Erst im Jahre 2010 erschien eine umfangreiche Studie von Ulrike Klöppel, die diesen historischen Ablauf analysierte und auch neuere Diskurse ab den 1950er-Jahren wissenschaftshistorisch in den Blick nahm [13].

Auffällig ist, dass sich im Gegensatz zur Frauenheilkunde, der Psychiatrie, der Rechtsmedizin oder Public Health Urologen und Kinderchirurgen sich erst relativ spät mit dem Themenkomplex auseinandersetzten [14, 15].

Während der Renaissance galten Wundergeburten („Monstrositäten“) als göttliche Zeichen und den Autoren war die Frage nach der Realität häufig zweitrangig. Einhorn, Pegasus oder Nixen fanden neben realen Lebewesen eine Darstellung in Abhandlungen über die Natur.

In der medizinhistorischen Literatur ist umstritten, wann genau (die meisten Autoren tendieren auf die Mitte bis das Ende des 18. Jahrhunderts) sich das zweigeschlechtliche dichotome Modell (nach Aristoteles) der Geschlechter gegenüber dem hippokratisch-galenischen mischgeschlechtlichen Modell durchsetzte [16, 17].

Ab der Aufklärung rückte die Sektion zur Analyse vermehrt in den Mittelpunkt des Interesses der Mediziner. Das Vorhandensein von Hoden und Penis galt als Zugehörigkeit zum männlichen Geschlecht, die Kriterien für das weibliche Geschlecht wurden häufig nicht beschrieben. Der Uterus wurde erst am Ende des 18. Jahrhunderts zu einem wichtigen Unterscheidungsmerkmal [18].

Auf juristischer Ebene schrieb 1756 der Bayrische Codex Maximilianeus Civils fest, die Geschlechtszuweisung durch einen Sachverständigen vornehmen zu lassen und das ganze Leben lang beizubehalten. Das preußische Allgemeine Landrecht von 1794, das bis zum Jahre 1900 in Kraft blieb, besaß eine analoge Regelung „Untersuchung durch Sachverständige“, die im Streitfall eingeholt werden sollte [19]. Hierauf wies der Kölner Rechtswissenschaftler Andreas Wacke bereits 1989 hin [20]. Bei Fragen der Ehefähigkeit und bei Scheidungsfällen infolge „impotentia generandi/impotentia coeundi“ spielten sie ebenfalls in der juristischen Literatur eine wichtige Rolle, wobei die wundärztliche Beseitigung von Ehehindernissen beschrieben wurde.

Mit Einführung der staatlichen Standesregister zum 1. Januar 1876 durch das Gesetz über die Beurkundung des Personenstandes und die Eheschließung vom 6. Februar 1875 in Preußen musste in das Geburtsregister das Geschlecht des Kindes zwingend eingetragen werden, wozu es ab 1899 ein entsprechendes Formular gab. Dieser Umstand begünstigte in der Folge Frühoperationen (s. unten) stark [21]. Erst im Jahre 2013 wurde diese Regelung abgeändert und der „Personenstandsfall“ konnte ohne Angabe eines Geschlechts („offen“) in das Geburtenregister eingetragen werden, wenn das Kind weder dem weiblichen noch dem männlichen Geschlecht zugeordnet werden konnte. Auch hatte dies zu Klagen von Betroffenen geführt. Erst seit dem 13.12.2018 kann neben männlich und weiblich die Entität „divers“ als positives Statement angegeben werden [22].

Im 19. Jahrhundert, mit Erstarken der naturwissenschaftlichen Medizin und einer entwicklungsphysiologischen Neubewertung der Gonaden [23], wurde der Hermaphrodismus neben Diskursen zur Homosexualität oder Transsexualität ein eigenständiges Feld [24–26].

**Figure 1:**
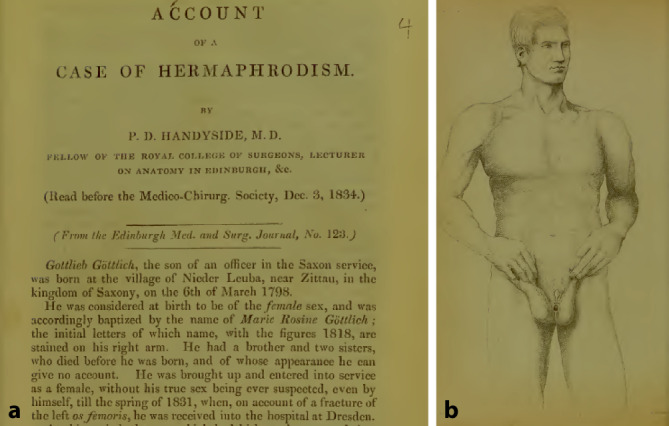

Erst ab dem beginnenden 20. Jahrhundert wurde dem „Geschlechtsempfinden“ der jeweiligen Personen in der Wissenschaft vermehrt Aufmerksamkeit geschenkt wie die Monographie von Franciszek Ludwik Neugebauer (1856–1914) oder Arbeiten von Magnus Hirschfeld (1868–1935) gut veranschaulichen (Abb. 1; [27, 28]).

Eine Form des Lebensunterhalts dieser Menschen bestand darin, sich von Wissenschaftlern gegen Entgelt im In- und Ausland untersuchen zu lassen. Gottlieb Göttlich/Marie Rosine Göttlich geb. 1798 aus Niederläuba bei Zittau wurde in Dresden, Göttingen (von Langenbeck d. Ä., Blumenbach), Heidelberg (Tiedemann), Bonn, Jena, Marburg, Mainz, Offenbach, Breslau, Bremen und Hamburg untersucht, sowie in Christiana, London, Dublin, Manchester, Liverpool, Cork, Dublin, Glasgow, Aberdeen, Montroose, Edinburgh. Initial als Mädchen erzogen, zog er ab 1832 Männerkleider an, nachdem Friedrich Tiedmann (1781–1861), Direktor des Anatomischen Instituts in Heidelberg, festgestellt hatte, dass es zu einem Descendus testis nach Leistenoperationen gekommen sei. Diese Diagnose war von Johann Friedrich Blumenbach (1752–1840) in Göttingen bestätigt worden.

Im ersten Drittel des 20. Jahrhunderts identifizierten Ärzte Hermaphroditen zunehmend als biologische „Gefahr“ für den „Volkskörper“. Sie ordneten Hermaphroditen als „minderwertig“ ein und stützten sich dabei auf ein Kontinuummodell des Geschlechts, das sich bereits um 1800 herausgeschält hatte. Die eugenische Problematisierung der „minderwertigen“ Geschlechtsentwicklung nahm in der Zeit des Nationalsozialismus deutlich zu, doch wurden Hermaphroditen nicht generell als erbkrank im Sinne des NS-Erbgesundheitsgesetzes eingestuft und einer Zwangssterilisierung zugeführt, doch waren sie häufig von Einzelfallentscheidungen der Erbgesundheitsgerichte abhängig [29].

Nach dem Zweiten Weltkrieg entwickelte sich das Primat geschlechtsangleichender Operationen in der frühen Kindheit („optimal gender policy“ nach Money [30]). Intersexverbände und Organisationen prangerten ab den 1990er-Jahren solche Eingriffe als Verstümmelung an sowie Verstöße gegen elementare Menschenrechte, da diese Operationen in einem Alter durchgeführt wurden, in dem die Kinder noch zu klein sind, um selbstständig darüber zu entscheiden. Auf medizinischer Ebene begann der Beginn des Umdenkens im Umgang mit Intergeschlechtlichkeit durch eine Konsensuskonferenz im Jahr 2005 in Chicago [31, 32]. Im Jahre 2012 empfahl der Deutsche Ethikrat [33], dass nicht Eltern oder Ärzte, sondern das Kind selber über sein Geschlecht entscheiden sollte – wenn es hierzu alt genug wäre. Auch andere Verbände raten inzwischen von rein kosmetischen Operationen ab und empfehlen Eingriffe nur noch dann, wenn sie medizinisch indiziert sind. „Von ärztlicher Seite ist inzwischen die Einsicht gewachsen, dass operative Eingriffe in der Tat bei einem mit einer Form von DSD-Neugeborenen nicht im Vordergrund stehen sollten und die Indikation in einem Kompetenzzentrum nach adäquater Diagnostik gestellt werden sollte“ (Krege; [34–36]).

## References

[1] Money J, Hampson JG, Hampson J (1955). An examination of some basic sexual concepts: the evidence of human hermaphroditism. Bull Johns Hopk Hosp.

[2] Gowan MJ (2012) Beratung und Intersexualität. Unterstützende Richtlinien für die Beratung von Angehörigen intersexueller Kinder. Diplomarbeit S 28–29, 30 Wien. https://core.ac.uk/download/pdf/11598981.pdf. Zugegriffen: 15. Jan. 2021

[3] Thaler C (2009) Being Intersex, Being Whole. In: IMPACT, Fall 2009. https://www.lambdalegal.org/sites/default/files/publications/downloads/impact_200910_being-intersex.pdf. Zugegriffen: 15. Jan. 2021

[4] Lang C (2006). Intersexualität-Menschen zwischen den Geschlechtern.

[5] Baumann H (1986). Das doppelte Geschlecht. Mythologien zur Bisexualität in Ritus und Mythos.

[6] Köppel U (2012) Medikalisierung „uneindeutigen“ Geschlechts APuZ 62 28–33. https://www.bpb.de/apuz/135440/medikalisierung-uneindeutigen-geschlechts. Zugegriffen: 15. Jan. 2021

[7] Shapiro S (1987). Amazons, hermaphrodites and plan monsters the “masculine” women in English satire and social criticism from 1580–1640. Atlantis.

[8] Ajootina A (1990). „Hermaphroditos“ Lexicon Iconographicum.

[9] Leroi AM (2008). Tanz der Gene: Von Zwittern, Zwergen und Zyklopen.

[10] Krämer F (2007). Die Individualisierung des Hermaphroditen in der Medizin und Naturgeschichte des 17. Jahrhunderts. Ber Wissenschaftsgesch.

[11] Foucault M (1991). Die Geburt der Klinik Eine Archäologie des ärztlichen Blicks.

[12] Foucault M, Defert D, Francouis E (2005). Polemik, Politik und Problematisierung. Schriften in vier Bänden.

[13] Klöppel U (2010). XXoXY0 ungelöst Hermaphrodismus, Sex und Gender in der deutschen Medizin Eine historische Studie zur Intersexualität.

[14] Hasche-Klünder R, Gelbke H, Anton HU (1958). Klinischer Beitrag zum Zwitterproblem. Z Urol.

[15] Young HH (1937). Genital abnormalties, hermaphrodism and related adrenal disorders.

[16] Stolberg M (2003). A woman down to her bones the anatomy of sexual difference in the sixteenth and early seventeenth centuries. Isis.

[17] Klöppel U (2010). XXoXY0 ungelöst Hermaphrodismus, Sex und Gender in der deutschen Medizin Eine historische Studie zur Intersexualität.

[18] Klöppel U (2010). XXoXY0 ungelöst Hermaphrodismus, Sex und Gender in der deutschen Medizin Eine historische Studie zur Intersexualität.

[19] ALR (1794) Allgemeines Landrecht für die Preussischen Staaten. Unter Andeutung der obsoleten oder aufgehobenen Vorschriften und Einschaltung der jüngeren noch geltenden Bestimmungen. Koch, Berlin, I 1 § 19–23

[20] Wacke A, Eyrich Heinz, Odersky W, Säcker F (1989). Vom Hermaphroditen zum Transsexuellen: Zur Stellung von Zwittern in der Rechtsgeschichte. Festschrift für Kurt Rebmann zum 65. Geburtstag.

[21] „Intersexualität“ Stellungnahme Deutscher Ethikrat. https://www.ethikrat.org/fileadmin/Publikationen/Stellungnahmen/deutsch/DER_StnIntersex_Deu_Online.pdf. Zugegriffen: 15. Jan. 2021

[22] Deutscher Bundestag (2018c) Drucksache 19/6477: Entwurf eines Gesetzes zur Änderung der in das Geburtenregister einzutragenden Angaben. http://dipbt.bundestag.de/dip21/btd/19/064/1906477.pdf. Zugegriffen: 22.03.2021

[23] Virchow R (1848). Der puerperale Zustand. Das Weib und die Zelle Gesammelte Abhandlungen zur wissenschaftlichen Medicin.

[24] Klöppel U (2010). ungelöst Hermaphrodismus, Sex und Gender in der deutschen Medizin Eine historische Studie zur Intersexualität.

[25] Mildenberger F, Stahnisch F, Steger F (2005). Diskursive Deckungsgleichheit. Hermaphroditismus und Homosexualität im medizinischen Diskurs (1850–1950). Medizin, Geschichte und Geschlecht. Körperhistorische Rekonstruktion von Identitäten und Differenzen.

[26] Stoff H (2004). Ewige Jugend. Konzepte der Verjüngung vom späten 19. Jahrhundert bis ins Dritte Reich.

[27] Neugebauer FL (1908). Der Hermaphrodismus des Menschen.

[28] Hirschfeld M (1923). Die intersexuelle Konstitution. Jahrb Sex Zwischenst.

[29] Klöppel U (2014) Intersex im Nationalsozialismus Ein Überblick über den Forschungsbedarf. In: Institut für Zeitgeschichte, Schwartz M (Hrsg) Homosexuelle im Nationalsozialismus. Neue Forschungsperspektiven zu Lebenssituationen von lesbischen, schwulen, bi-, trans- und intersexuellen Menschen 1933 bis 1945. De Gruyter Oldenbourg, München, S 107–114 (https://sexualityandholocaust.files.wordpress.com/2018/10/klocc88ppel_intersex.pdf Zugegriffen 15.11.2020)

[30] Money J, Hampson JG, Hampson JL (1955). Hermaphroditism: Recommendations concerning assignment of sex, change of sex, and psychological management. Bull. Johns. Hopkins Hosp..

[31] Hughes IA, Houk C, Ahmed SF, Lee PA, LWPES1/ESPE2Consensus Group (2006). Consensus statement on management of intersex disorders. J Pediatr Urol.

[32] Schweizer K, Köster E-M, Richter-Appelt H (2019). Varianten der Geschlechtsentwicklung und Personenstand Zur „Dritten Option“ für Menschen mit intergeschlechtlichen Körpern und Identitäten. Psychotherapeut.

[33] „Intersexualität“ Stellungnahme Deutscher Ethikrat. https://www.ethikrat.org/fileadmin/Publikationen/Stellungnahmen/deutsch/DER_StnIntersex_Deu_Online.pdf. Zugegriffen: 15. Nov. 2020

[34] Krege S (2020). Reformen in der Behandlung von Menschen mit Varianten der Geschlechtsdifferenzierung. Urologe.

[35] S2k-Leitlinie „Varianten der Geschlechtsentwicklung“, AWMF-Register Nr. 174/001

[36] Ludwikowski B, Krege S (2020). Genitalchirurgie im Spiegel gesellschaftspolitischer Entwicklungen. Uro-News.

